# The “root” causes behind the anti-inflammatory actions of ginger compounds in immune cells

**DOI:** 10.3389/fimmu.2024.1400956

**Published:** 2024-06-28

**Authors:** Kitti Pázmándi, Attila Gábor Szöllősi, Tünde Fekete

**Affiliations:** Department of Immunology, Faculty of Medicine, University of Debrecen, Debrecen, Hungary

**Keywords:** ginger, gingerol, shogaol, immune cell, cytokine, signaling

## Abstract

Ginger (*Zingiber officinale*) is one of the most well-known spices and medicinal plants worldwide that has been used since ancient times to treat a plethora of diseases including cold, gastrointestinal complaints, nausea, and migraine. Beyond that, a growing body of literature demonstrates that ginger exhibits anti-inflammatory, antioxidant, anti-cancer and neuroprotective actions as well. The beneficial effects of ginger can be attributed to the biologically active compounds of its rhizome such as gingerols, shogaols, zingerone and paradols. Among these compounds, gingerols are the most abundant in fresh roots, and shogaols are the major phenolic compounds of dried ginger. Over the last two decades numerous *in vitro* and *in vivo* studies demonstrated that the major ginger phenolics are able to influence the function of various immune cells including macrophages, neutrophils, dendritic cells and T cells. Although the mechanism of action of these compounds is not fully elucidated yet, some studies provide a mechanistic insight into their anti-inflammatory effects by showing that ginger constituents are able to target multiple signaling pathways. In the first part of this review, we summarized the current literature about the immunomodulatory actions of the major ginger compounds, and in the second part, we focused on the possible molecular mechanisms that may underlie their anti-inflammatory effects.

## 1 Introduction

The rhizome of ginger (*Zingiber officinale*) has been used as a dietary food supplement across China and Southeast Asia since ancient times. Ginger was first documented as an herbal medicine around 3000 BC in China (1). It was primarily recommended as a remedy for cold, fever, leprosy and tetanus (1). Other medicinal uses of ginger include the treatment of nausea, upset stomach and it’s use as a digestive aid (2). The U.S. Food and Drug Administration (FDA) classified ginger root as a safe herbal supplement that can be used in complementary and alternative medicine preparations (3). Due to its proven beneficial effects, in 2012 the European Medicines Agency listed the ginger rhizome as an approved treatment modality in the prevention of nausea and vomiting associated with motion sickness (4). In the last few years a myriad of studies indicated that ginger possesses various biological activities such as anti-inflammatory, antioxidant, anti-microbial, anti-cancer and neuroprotective effects. It has also been revealed that the pharmacological benefits of ginger can be credited to the bioactive compounds of its rhizome.

**Figure 1:**
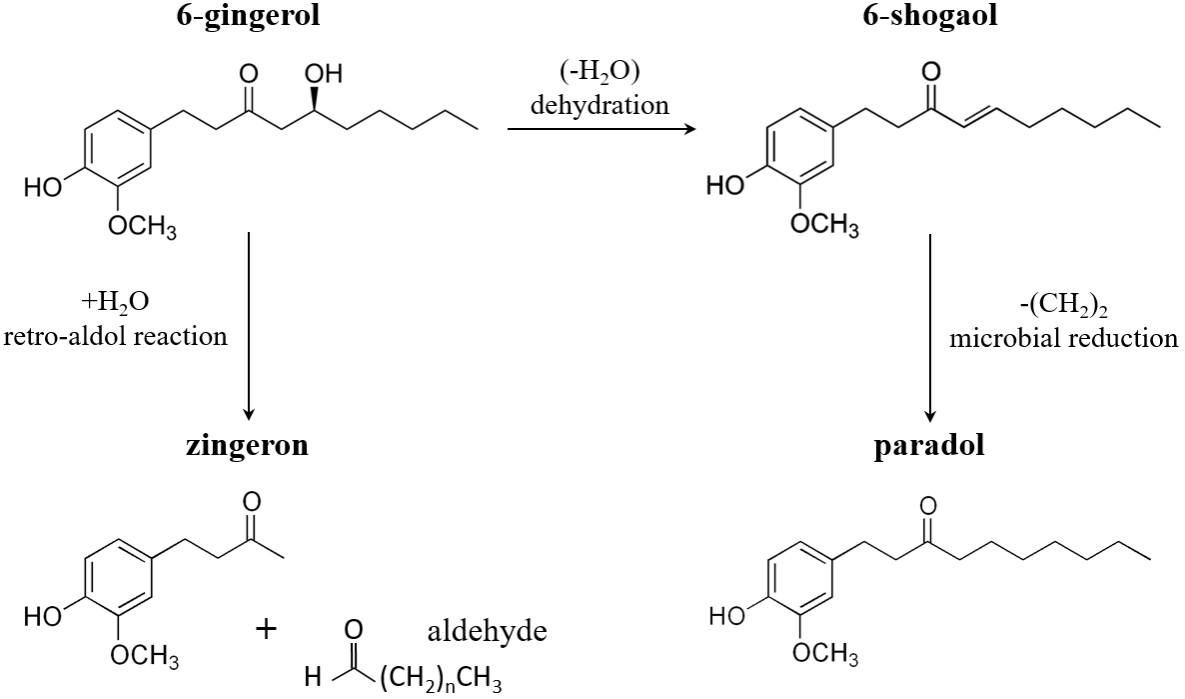
Conversion of 6-gingerol into 6-shogaol, paradol and zingerone. Upon heating or drying 6-gingerol is mostly converted to 6-shogaol through dehydration and to a lesser extent to zingerone through a retro-aldol reaction. Moreover, 6-shogaol is transformed to 6-paradol by microbial metabolism.

To date, more than 400 chemical compounds such as lipids, terpenes, carbohydrates and phenolic compounds have been identified in the ginger rhizome (5, 6). The phenolic compounds or phenolics, which are usually referred to as the nonvolatile components of ginger, are mainly responsible for its pharmacological activities and consist of gingerols, shogaols, paradols and zingerone (7). Gingerols are the main pungent components of fresh ginger that can be differentiated based on their unbranched alkyl side chain length. Among them, 6-gingerol is the most abundant constituent that is followed by 8-gingerol and 10-gingerol (8). Upon drying or heating of ginger root the thermally labile gingerols undergo dehydration reactions to form the corresponding shogaols, which are twice as pungent as gingerols (9). Although shogaols are scarcely found in fresh ginger root, 6-shogaol is the predominant bioactive compound in the dried rhizome. At high temperatures or by microbial metabolism shogaols might be partly transformed to paradols, which represent a minor but important bioactive constituent of ginger (10). Cooking or drying can also convert gingerol into zingerone through a retro-aldol reaction (11). In addition, a number of other gingerol derivatives such as gingerdion, gingerdiol, dehydrogingerdione and other minor components have been isolated from ginger rhizome (12). A recent study using synthetic strategy to prepare 6-gingerol derivatives indicates that several of those show promising anti-platelet and antioxidant activities; however, their biological effects needs to be further elucidated (13). The major bioactive compounds of ginger and the conversion of 6-gingerol into 6-shogaol, paradol and zingerone are shown in **Figure 1**.

In the last two decades, especially since the outbreak of coronavirus disease 2019 (Covid-19) pandemic, herbal plants have gained special interest as an alternative or complementary medicine due to their beneficial medicinal properties and minimal adverse effects (14, 15). Ginger is one of the most researched medicinal plants due to its long-known health benefits and high bioactive agent content. In the last few years, a number of studies have proven the pharmacological potential of ginger-derived phytochemicals; however, the cellular and molecular mechanisms behind their activities are not fully revealed yet. It was shown that ginger phytochemicals might exert their anti-inflammatory effects by modulating the function of various immune cells such as macrophages, neutrophils, T cells and dendritic cells (DC) (5). It was also demonstrated that ginger constituents have the potential to regulate key signaling pathways such as the nuclear factor-kappa B (NF-κB), mitogen activated protein kinase (MAPK) and phosphatidylinositol-3-kinase (PI3K)/Akt/mammalian target of rapamycin (mTOR) signaling cascades in these cell types. Some studies also provided a deeper insight into the mechanism of action of ginger constituents by showing that those are able to target several signaling molecules such as the AMP-activated protein kinase (AMPK), nuclear factor erythroid 2-related factor 2 (NRF2), heme oxygenase-1 (HO-1), and peroxisome proliferator-activated receptor gamma (PPARγ).

Although ginger rhizome is abundant in bioactive compounds, the *in vitro* and *in vivo* studies mainly focus on gingerols and shogaols, since those possess the most significant pharmacological effects. Some reports also suggest that shogaols exhibit more potent biological activities then gingerols (16). In particular, the loss of the hydroxyl group in their sidechains might increase their lipophilicity and thus shogaols show a superior bioavailability compared to gingerols (9). Reviews published in the last few years focused on the general biological effects (5), anti-cancer (17, 18) or neuroprotective (19) activities of ginger constituents. Some reports also addressed the anti-inflammatory actions of ginger extract, or one or the other components in general (20, 21). Nevertheless, the current literature lacks a comprehensive review on the immunomodulatory effects of ginger phytochemicals at the cellular and molecular level. Most importantly, an update is needed to discuss the scientific merit of the most recently published research articles. Therefore, in this recent review we aimed to collate all the available evidence on the effects of the major ginger constituents on different immune cell types and to highlight the latest advances in our understanding of their mechanisms of anti-inflammatory actions.

## 2 Effects of ginger compounds on the cells of the immune system

Ginger has been used for centuries for its anti-emetic effect. Recent clinical trials demonstrated that ginger supplementation can be used as an adjuvant therapy for managing and preventing chemotherapy-induced nausea and vomiting as well (22, 23). Additionally, some clinical studies also suggested that ginger can effectively ameliorate chronic pain in conditions such as arthritis (24, 25). Interestingly, the pain-relieving properties of ginger constituents are similar to that exerted by the non-steroidal anti-inflammatory drugs such as ibuprofen (26, 27). Research data also indicates that the pain reducing effect of ginger is linked to its high anti-inflammatory capacity [reviewed in (28)]. Indeed, a number of *in vitro* and *in vivo* studies demonstrated that the bioactive compounds of ginger are able to dampen the inflammatory responses of various immune cells. Most of these studies focused on the innate immune machinery; however, there are a few reports available on the effects of the major ginger compounds on T cells as well. This chapter summarizes those studies that investigated the *in vitro* effects of ginger phenolics on different immune cell types.

### 2.1 Effects of ginger phenolics on macrophages

Macrophages serve as the first line of defense against invading pathogens and represent a highly plastic cell population with divergent phenotypes and functions. Generally, macrophages play an essential role in clearing out microbial pathogens by producing antimicrobial molecules such as reactive oxygen species (ROS) and nitric oxide (NO). In response to pathogenic stimuli macrophages also secrete various types of cytokines and chemokines and thus contribute to the initiation of inflammatory responses. Owing to their high functional plasticity, macrophages are also involved in the resolution of inflammation and restoration of homeostasis (29). Nevertheless, abnormal activation and polarization of macrophages has been suggested to contribute to the pathogenesis of different autoimmune diseases (30).

The very first study investigating the effects of ginger components on immune cells was published in 2003. Ippoushi et al. demonstrated that 6-gingerol decreased the production of NO and the protein levels of inducible NO synthase (iNOS) in lipopolysaccharide (LPS)-stimulated J774.1 murine macrophages (31). 6-gingerol also protected against DNA and protein damage by suppressing the peroxynitrite-induced single strand breaks in supercoiled plasmid DNA as well as the formation of nitrotyrosine in these cells (31). In mouse peritoneal macrophages, 6-gingerol decreased the LPS-triggered production of the inflammatory cytokines interleukin 1 beta (IL-1β), tumor necrosis factor (TNF), IL-12 and the chemokine CCL5 (RANTES), whereas it had no effect on the upregulation of major histocompatibility complex (MHC) class II, and the co-stimulatory molecules CD80 (B7.1) and CD86 (B7.2) (32). The authors also gave some mechanistic insight by demonstrating that 6-gingerol was able to suppress the LPS-induced activation of NF-κB (32). In the human monocytic U937 cell line, 6-, 8-, 10-gingerols efficiently reduced the mRNA level of cyclooxygenase-2 (COX-2), a key enzyme responsible for prostaglandin E2 (PGE_2_) production, and thus were more potent inhibitors of PGE_2_ production compared to 6-shogaol (33). On the contrary, 6-shogaol more effectively decreased the level of PGE_2_, COX-2 and iNOS than 6-gingerol in LPS-stimulated murine RAW 264.7 cells (34). The authors further demonstrated that 6-shogaol blocked the LPS-mediated phosphorylation and degradation of inhibitor kBα (IκBα) that suppressed the subsequent phosphorylation, nuclear translocation and transcriptional activity of NF-κB p65 in RAW 264.7 cells. Furthermore, 6-shogaol reduced the activation of PI3K and Akt and interfered with the MAPK signaling pathway by attenuating the phosphorylation of extracellular-regulated kinase 1/2 (ERK1/2) but not that of p38 in LPS-stimulated RAW 264.7 macrophages (34). Similarly, another study demonstrated that 6-gingerol inhibited iNOS and TNF production through the suppression of IκBα phosphorylation and NF-κB activation (35). It was also shown that 6-gingerol inhibits the LPS-triggered intracellular Ca^2+^ mobilization and ROS generation, most likely by blocking the cytosol-to-membrane translocation of protein kinase C-α (PKC-α) (35).

In LPS-stimulated primary microglia cells, 6-shogaol significantly decreased NO levels by reducing iNOS activity, suppressed PGE_2_ production by downregulating COX-2 expression, and reduced IL-1β and TNF secretion by inhibiting IκBα phosphorylation and degradation, and thereby NF-κB activation (36). Nevertheless, in contrast to the results obtained by RAW264.7 macrophages (30), 6-shogaol inhibited the LPS-stimulated activation of p38 and JNK but not that of ERK1/2 in primary microglia cells (32). A few years later similar results were published showing that 6-shogaol suppressed LPS-induced IL-1β, IL-6, TNF and PGE_2_ production by inhibiting the phosphorylation and nuclear translocation of NF-κB in BV2 cells (37). As a new concept the authors suggested that 6-shogaol blocks the LPS-induced inflammatory mediator production through activating PPARγ, which is a known inhibitor of NF-κB activation (33). Similar to 6-shogaol, 6-gingerol also effectively inhibited the LPS-stimulated expression of iNOS and production of NO, IL-1β and IL-6 in primary mouse microglia (38). Furthermore, 6-gingerol was able to suppress the phosphorylation of Akt, mTOR and signal transducer and activator of transcription 3 (STAT3), the latter of which is a crucial signaling intermediate for TLR4-induced inflammatory responses in macrophages (39).

In primary mouse calvarial osteoblasts, 6-shogaol also inhibited the IL-1-induced expression of PGE_2_ by suppressing the enzymatic activity of COX-2 and PGE synthase that resulted in decreased receptor activator of NF-κB (RANKL) production and thus reduction of osteoclast differentiation (40).

Finally, two reports investigated the effect of gingerols and shogaols on the nucleotide-binding oligomerization domain-like receptor family pyrin domain containing 3 (NLRP3) inflammasome-mediated responses. The first study compared the inhibitory effects of 6-, 8-, 10-gingerols and 6-, 8-, 10- shogaols in human THP-1 macrophages stimulated with LPS and adenosine 5’-triphosphate (ATP) (41). It was demonstrated that 10-gingerol and all the shogaols effectively reduced the secretion of TNF and IL-1β as well as the protein levels of NLRP3 and caspase-1. In general, shogaols show a higher anti-inflammatory capacity than the corresponding gingerols that could be attributed to the α, β-unsaturated carbonyl group in the structure of shogaols. Among the investigated phytochemicals 6-shogaol was the most potent inhibitor of NLRP3-mediated inflammasome activation. Interestingly, the results also indicate that the increase in alkyl side chain length weakens the anti-inflammatory potential of shogaols, while enhancing that of gingerols (41). The other study was conducted on RAW 264.7 cells and mouse bone marrow-derived macrophages (BM-DMs), in which 6-gingerol greatly reduced the release of caspase-1 p20 as well as the production of IL-1β and IL-18 in response to ATP and LPS (42). 6-gingerol significantly suppressed the phosphorylation of p38, JNK and ERK1/2 in RAW 264.7 cells, while it inhibited only ERK1/2 activation in BM-DMs. Treatment of RAW 264.7 cells with an ERK agonist reversed the inhibitory effects of 6-gingerol on caspase-1 p20 release suggesting that 6-gingerol exerts its effect through blocking MAPK activation (42).

Recent *in vivo* studies further suggest that 6-gingerol might inhibit NLPR3 inflammasome activation by reducing oxidative stress. In the lung tissues of LPS-subjected rats, 6-gingerol repressed the levels of oxidative stress markers and the expression of NLRP3 inflammasome (43). 6-gingerol also decreased the arsenic-trioxide induced production of ROS and the expression of NLRP3 inflammasome components in the liver tissues of mice (44). These current findings indicate that 6-gingerol migth exert its anti-inflammatory effects by inhibiting the ROS-NLRP3 inflammasome pathway.

**Figure 2:**
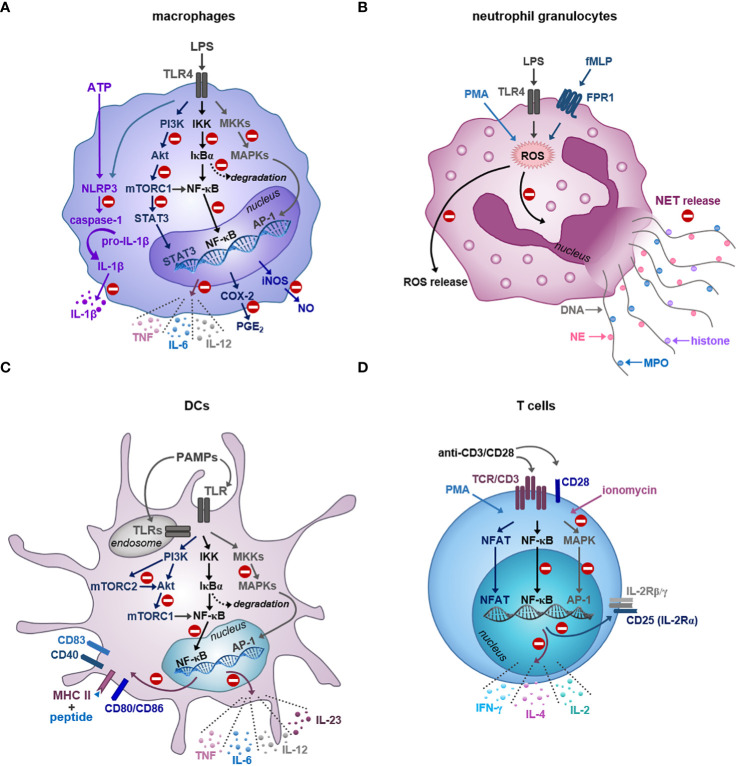
Inhibition of immune cell functions by the bioactive compounds of ginger. **(A)** Gingerols and shogaols reduce the LPS-triggered production of inflammatory cytokines, PGE_2_ and NO by interfering with the activation of NF-κB, MAPK and mTOR signaling pathways at different levels in macrophages. Ginger phenolics also inhibit the NLRP3-mediated inflammasome activation and subsequent production of IL-1β in macrophages. **(B)** Ginger phytochemicals display antineutrophil potential by effectively inhibiting ROS generation and netosis in response to various stimuli. **(C)** Similar to macrophages, ginger-derived compounds also attenuate human DC functionality via suppressing the NF-κB, MAPK and mTOR signaling pathways at various levels. Consequently, the bioactive compounds of ginger are able to reduce the cytokine production, costimulatory molecule expression and T cell stimulatory ability of DCs. **(D)** Gingerols are also potent inhibitors of T cell activation, proliferation and polarization. The regulatory action of ginger phenolics is indicated by the minus symbol. *MKK: MAPK kinase; NFAT: nuclear factor of activated T cells*.

**Table 1** summarizes the effects of the major ginger compounds on macrophages, whereas **Figure 2A** shows the signaling pathways, which are affected by the regulatory actions of major ginger phenolics in these cells.

**Table 1:** Effects of the major ginger phenolics on macrophages.

Macrophages					
Compounds	Cell types	Challenges	Observed effects	Potential mechanisms	Ref.
6-gingerol	J774.1 murine macrophage cell line	LPS	• reduced NO, iNOS levels • protection against DNA and protein damage	–	(31)
6-gingerol	mouse peritoneal macrophage	LPS	• decreased IL-1β, IL-12, TNF, CCL5	• suppressed NF-κB activity	(32)
6-, 8-, 10-gingerols and 6-shogaol	human monocytic U937 cell line	LPS	• reduced PGE_2_ production • gingerols reduced COX-2 expression	–	(33)
6-shogaol	RAW 264.7 murine macrophages cell line	LPS	• reduced PGE_2_ production • reduced COX-2, iNOS levels	• blocked IκBα phosphorylation and degradation • suppressed NF-κB activity • inhibited PI3K/Akt and ERK1/2 activation	(34)
6-gingerol	RAW 264.7 murine macrophages cell line	LPS	• inhibited iNOS and TNF expression • reduced intracellular Ca^2+^ mobilization • decreased ROS generation	• suppressed IκBα phosphorylation, NF-κB nuclear translocation and PKC-α translocation	(35)
6-shogaol	primary microglia	LPS	• reduced IL-1β, TNF and PGE_2_ production • downregulated COX-2 expression	• blocked IκBα phosphorylation and nuclear translocation • decreased NF-κB activity • inhibited p38 and JNK activation	(36)
6-shogaol	BV2 murine microglial cell line	LPS	• suppressed IL-1β, IL-6, TNF and PGE_2_ production	• reduced NF-κB phosphorylation and nuclear translocation • increased PPARγ activity	(37)
6-gingerol	primary mouse microglia cell	LPS	• reduced NO, iNOS levels • reduced IL-1β and IL-6 production	• reduced Akt, mTOR and STAT3 phosphorylation	(38)
6-shogaol	primary mouse calvarial osteoblast	IL-1α	• reduced PGE_2_ expression • decreased RANKL production and osteoclast differentiation	• suppressed COX2 and PGE_2_ synthase activity	(40)
6-, 8-, 10-gingerols/shogaols	THP-1 human monocytic cell line	LPS+ATP	• reduced TNF and IL-1β secretion • reduced NLRP3 and caspase-1 levels	–	(41)
6-gingerol	RAW 264.7 macrophage cell line and mouse BM-DM	LPS+ATP	• reduced IL-1β and IL-18 secretion • reduced caspase-1 p20 release	• suppressed MAPK activation	(42)

### 2.2 Effects of the major ginger phenolics on neutrophil granulocytes

As phagocytic cells of the innate immune system, neutrophil granulocytes take an important part in clearing infectious agents and cellular debris from the human body. In addition to their phagocytic capacity, activated neutrophils extrude neutrophil extracellular traps (NETs), composed of DNA, histones, and antimicrobial enzymes, such as neutrophil elastase (NE) and myeloperoxidase (MPO), in which pathogens are immobilized and exposed to a lethal dose of effector proteins (45). A large body of evidence also indicates that through NET release neutrophils are implicated in the pathogenesis of autoimmune diseases such as lupus (46).

It was first published in 2010 that 6-,8-,10-gingerols and 6-shogaol are able to effectively inhibit ROS generation by human neutrophils in response to formylmethionine-leucyl-phenylalanine (fMLP), a strong inducer of neutrophil activation (16). Among the tested ginger phenolics, 6-shogaol showed the highest potential to suppress fMLP-induced ROS release, while 6-gingerol had the weakest inhibitory capacity (16). More than 10 years later, a study from 2021 also investigated the anti-inflammatory properties of gingerols in neutrophils. Ali et al. demonstrated that 6-, 8-, and 10-gingerols suppressed NET-associated MPO levels, thereby netosis of human neutrophils in response to LPS, phorbol 12-myristate 13-acetate (PMA) and various lupus-relevant stimuli as well (47). All three gingerols suppressed the formation of hydrogen-peroxide (H_2_O_2_) in neutrophils indicating that gingerols attenuate netosis by inhibiting the generation of ROS. Further, 6-gingerol also decreased the activity of phosphodiesterase 4 (PDE4), which by regulating cAMP levels plays a prominent anti-inflammatory effect in basically all cells involved in inflammation (48). Concomitantly, 6-gingerol increased the intracellular levels of cAMP and thus that of cAMP-dependent protein kinase A (PKA), which exerts anti-inflammatory activity by promoting the production of anti-inflammatory cytokines and by reducing the transcriptional activity of NF-κB (48). Mechanistically, the authors proposed that the antineutrophil effects of 6-gingerol depend, at least partially, on its ability to inhibit PDE4 activity (47).

A year later it was published that zingerone, a less-studied component of ginger, also displays antineutrophil potential (49). In PMA-stimulated mouse bone marrow-derived neutrophils, zingerone significantly suppressed ROS production and formation of NET, as indicated by the decreased level of DNA-associated MPO. Interestingly, the bactericidal activity of neutrophils was not affected by zingerone treatment either *in vitro* or *in vivo*. In particular, zingerone did not alter the phagocytic capacity of neutrophils *in vitro* and had no effect on bacteria dissemination *in vivo*. Nevertheless, zingerone treatment significantly increased the levels of the nuclear NRF2 and HO-1 in PMA-stimulated neutrophils. Further it was shown that a specific inhibitor of NRF2 could efficiently reverse the protective effect of zingerone in septic mice (49).

In contrast with the previous findings, a recent study reported that 6-gingerol increased fMLP-stimulated CXCL8 chemokine secretion and ROS production in primary human neutrophils (50). Furthermore, 6-gingerol also increased the expression of neutrophil surface markers such as CD11b and CD66, as well as the expression of formyl peptide receptor 1 (FPR1), which may lead to increased responsiveness to its ligand, fMLP. In this particular study, the authors used a very low concentration of 6-gingerol (50 nM), which is generally a hundred- and thousand-fold lower compared to the doses used in previous publications on neutrophils and other immune cell types. The report also suggested that 6-gingerol applied in a low dose facilitates neutrophil functions through binding to the transient receptor potential cation channel subfamily V member 1 (TRPV1), the potential of which as a mechanism of action we discuss in chapter 4.4 in detail.

The effects of the major ginger phenolics on neutrophil granulocytes are included in **Table 2**, whereas their antineutrophil actions are shown in **Figure 2B**.

**Table 2:** Effects of the major ginger phenolics on neutrophil granulocytes.

Neutrophil granulocytes					
Compounds	Cell types	Challenges	Observed effects	Potential mechanisms	Ref.
6-,8-,10-gingerols and 6-shogaol	human neutrophil	fMLP	• suppressed ROS release	–	(16)
6-, 8-, and 10-gingerol	human neutrophil	LPS, PMA	• suppressed netosis • reduced ROS generation	• decreased PDE4 activity • increased cAMP levels and PKA	(47)
zingerone	mouse BM-derived neutrophil	PMA	• suppressed ROS production • reduced netosis	• activated the NRF2 signaling pathway	(49)
6-gingerol	human neutrophil	fMLP	• increased ROS and CXCL8 production • increased CD11b, CD66, FPR1 expression	• binding to TRPV1	(50)

### 2.3 Effects of 6-gingerol and 6-shogaol on DCs

DCs represent a heterogeneous population, which acting as the most potent professional antigen presenting cells (APCs) bridge the innate and adaptive immune systems. Expressing a wide repertoire of innate receptors, DCs can recognize a broad range of pathogen-, and danger-associated molecular patterns (PAMPs and DAMPs), and then migrate to secondary lymphoid organs to present antigens to T cells. Activated DCs upregulate costimulatory molecules and produce polarizing cytokines to drive T cell differentiation, and initiate different types of antigen-specific adaptive immune responses. In the steady-state, in the absence of activation signals, antigen presentation by DCs leads to T cell unresponsiveness and tolerance (51). In addition to their well-known functions of controlling innate and adaptive immunity, accumulating evidence indicates that DCs are implicated in the pathogenesis and pathomechanism of various autoimmune disorders (52).

Although DCs play a central role in the coordination of immune responses, only two studies investigated how ginger-derived phytochemicals might affect their functionality. Han et al. studied the effects of 6-gingerol on mouse BM-DCs, and found that 6-gingerol was able to remarkably reduce the production of TNF, IL-1β, IL-6 and IL-23 as well as the expression of CD80, CD86 and MHC II in response to LPS exposure (53). 6-gingerol treated BM-DCs also had an inferior capacity to prime T helper 17 (Th17) cell polarization upon co-culture with CD4+ naïve T cells. Further, it was demonstrated that 6-gingerol prevented the LPS-induced phosphorylation of NF-κB, JNK and ERK1/2 suggesting that 6-gingerol suppressed the activation of BM-DCs by interfering with the NF-κB and MAPK signaling cascades (53).

Our research group has recently reported that 6-gingerol and 6-shogaol are also able to modulate the phenotypical and functional properties of human DCs (54). The studies so far have investigated the effect of 6-gingerol or 6-shogaol on the TLR4-mediated response of immune cells. In our experiments, monocyte-derived DCs (moDCs) were first treated with 6-gingerol and 6-shogaol then were exposed to various TLR ligands including agonists of TLR4, TLR2/1 and TLR7/8. We found that 6-gingerol and 6-shogaol could significantly decrease the TLR-triggered expression of CD40, CD83, CD86 and HLA-DQ in moDCs. Furthermore, 6-gingerol and 6-shogaol significantly reduced the secretion of TNF, IL-6 and IL-10 by TLR-stimulated moDCs. Similarly, both compounds significantly reduced the *Escherichia coli*-triggered secretion of inflammatory cytokines, and thus their capacity to promote a Th1 phenotype in naïve CD4+ T cells (54). Investigating the mechanism underlying the actions of these compounds, we found that both 6-gingerol and 6-shogaol could significantly decrease the TLR-induced nuclear translocation of NF-κB p65, while not affecting IκBα degradation. Both compounds were also able to reduce the TLR-triggered activation of the MAPK pathway. Further, we demonstrated that 6-gingerol decreased mTOR complex 2 (mTORC2)-mediated Akt phosphorylation, whereas it had no effect on mTORC1-mediated p70S6K phosphorylation in TLR-stimulated moDCs. In contrast, 6-shogaol could greatly suppress the TLR-mediated activation of both mTOR complexes. Further, we found that 6-shogaol but not 6-gingerol could significantly increase the activity of AMPK, the nuclear translocation of NRF2 and the expression of HO-1. Altogether, our data imply that both ginger-derived compounds attenuate human DC functionality via suppressing the NF-κB, MAPK and mTOR signaling pathways. Further, we demonstrated that compared to 6-gingerol, 6-shogaol possesses a stronger inhibitory activity on moDCs that might be attributed to its ability to activate AMPK and the NRF2/HO-1 system (54).

The effects of 6-gingerol and 6-shogaol on DCs are summarized in **Table 3**, and illustrated in **Figure 2C**.

**Table 3:** Effects of 6-gingerol and 6-shogaol on DCs.

DCs					
Compounds	Cell types	Challenges	Observed effects	Potential mechanisms	Ref.
6-gingerol	mouse BM-DC	LPS	• reduced TNF, IL-1β, IL-6 and IL-23 secretion • reduced CD80, CD86 and MHC II expression • reduced Th17 priming capacity	• inhibited NF-κB, JNK and ERK1/2 activation	(53)
6-gingerol, 6-shogaol	human moDC	various TLR ligands	• decreased costimulatory molecule and MHC II expression • reduced TNF, IL-6 and IL-10 production • decreased capacity to prime Th1 T cells	• inhibited NF-κB nuclear translocation • reduced mTOR and MAPK activity • 6-shogaol increased AMPK and NRF2 activity	(54)

### 2.4 Effects of gingerols on T cells

As specialized cells of the adaptive immune system, T cells play a central role in directing immune responses against pathogens, allergens, or tumors. While CD4+ T helper cells modulate immune responses by facilitating the activity of other immune cells, regulatory T cells (Treg) contribute to the maintenance of immune homeostasis and CD8+ cytotoxic T cells are essential to the elimination of cancer cells and virally infected cells (55). Nevertheless, it must be noted that specific populations of T cells such as Th17 cells might contribute to the pathogenesis of various chronic inflammatory disorders (56).

The first study regarding the effects of gingerols on the activation and effector function of T cells was published in 2015. Bernard et al. found that 6-, 8- and 10-gingerols inhibited the proliferation of mouse spleen-derived CD3+ T cell in response to anti-CD2/CD3/CD28-coated beads (Dynabeads) or syngeneic DCs (57). 8- and 10-gingerols also significantly decreased the expression of the activation markers CD25 and CD69. All three gingerols reduced the Dynabead-stimulated production of interferon-gamma (IFN-γ), but did not affect IL-4 synthesis. Moreover, 8- and 10- gingerols decreased IL-2 secretion as well. In general, the data suggest that 8- and 10-gingerols are more potent inhibitors of T cell activation and proliferation than 6-gingerol (57).

In accordance with the above, another study demonstrated that 6-gingerol is able to suppress the production of IFN-γ and IL-4 by mouse spleen-derived CD4+ T cells cultured under Th1 or Th2 polarizing conditions, respectively (58). 6-gingerol also significantly suppressed the proliferation of pan T cells isolated from ovalbumin (OVA)-sensitized mice. In addition, 6-gingerol abrogated the staphylococcal enterotoxin B (SEB)-induced proliferation of unprimed T cells and the proliferation of human Jurkat T cells in response to anti-CD3/CD28 and IL-2. Pre-treatment with 6-gingerol also inhibited the activation of p38, ERK1/2 and JNK, as well as the nuclear translocation of NF-κB and c-fos transcription factors in Jurkat cells stimulated with PMA and ionomycin. Thus, the results suggest that 6-gingerol suppresses T cell activation and proliferation through inhibiting NF-κB and activating protein 1 (AP-1) activation (58).

The effects of 6-gingerol on CD8+ T cells were studied only in the context of cancer. Two publications reported that 6-gingerol is able to increase the number of tumor-infiltrating CD8+ T cell in mice that might play an important role in the anti-tumor effect of ginger (59, 60). The anti-cancer activity of ginger compounds is not in the scope of the recent review; however, it has been discussed in detail elsewhere (17, 18). The effects of gingerols on CD4+ T cells are summarized in **Table 4**, and illustrated in **Figure 2D**.

**Table 4:** Effects of gingerols on T cells.

T cells					
Compounds	Cell types	Challenges	Observed effects	Potential mechanisms	Ref.
6-, 8- and 10-gingerols	mouse spleen-derived CD3+ T cell	Dynabead, syngeneic DCs	• decreased proliferation • reduced expression of CD25 and CD69 • reduced IFNγ and IL-2	–	(57)
6-gingerol	mouse spleen-derived CD4+ T cell	ovalbumin, Th1 or Th2 polarizing conditions, SEB	• decreased proliferation • reduced IFNγ and IL-4 production	–	(58)
6-gingerol	Jurkat human T cell line	PMA, ionomycin anti-CD3/CD28 and IL-2	• reduced proliferation • decreased T cell activation	• inhibited MAPK activation • suppressed NF-κB and AP-1 activation	(58)

In conclusion, the above data indicate that the bioactive compounds of ginger can efficiently modulate the functionality of different immune cell types, mostly via interfering with the activity of various signaling pathways in them. Nevertheless, opposing data were also published regarding the effects of different ginger phenolics that might be attributed to the varying cell types, culture conditions and stimuli applied in these studies. Furthermore, the effect of ginger phenolics on other immunce cell types such as NK cells, basophils, and eosinophils have not been explored yet. Furthermore, in lymphomatic cell lines with B cell origin only the anti-cancer potential of these compounds were investigated (61, 62).

Altogether, the pleiotropic effects of ginger phenolics suggest that their mechanism of action likely involves multiple regulatory mechanisms, which is discussed in chapter 4 in details.

## 3 Protective effects of ginger phenolics in animal models of autoinflammatory and autoimmune diseases

Abnormal activation of the immune system can lead to the generation of autoinflammatory or autoimmune disorders. The pathogenesis of autoimmune diseases is characterized by loss of tolerance against self-tissues, appearance of autoreactive T and B cells, and production of autoantibodies. On the contrary, autoinflammatory diseases are mainly caused by altered innate immunity. They are also characterized by the activation of inflammasomes, and lack of self-reactive antibodies and T cells (63). Although there is a wide range of treatment options to manage symptoms, currently there is no cure for these disorders. As we introduced above several reports suggest that gingerols and shogaols might also alleviate disease symptoms due to their strong antioxidant and anti-inflammatory activities. These assumptions were later also supported by data from animal models of inflammation such as sepsis (42, 64, 65) or neuroinflammation (36, 38). Here we summarize the most recent *in vivo* animal data on the efficacy of gingerols and shogaols in the treatment of autoinflammatory and autoimmune diseases.

Ulcerative colitis (UC), one of the main forms of inflammatory bowel disease, is characterized by chronic recurrent inflammation of the large intestine. The etiology and pathogenesis of these diseases is not yet fully understood, although it appears to involve both autoinflammatory and autoimmune traits (66). A study investigated the therapeutic efficacy of intraperitoneally-injected 6-, 8-, and 10-gingerols in a dextran sulfate sodium (DSS)-induced rat colitis model (67). Results show that all three gingerols attenuated DSS-induced symptoms of colitis and accelerated the healing of mucosal damage. Gingerols elevated the activity of the anti-inflammatory enzyme superoxide dismutase (SOD), while reduced the activity of MPO, a marker of neutrophil infiltration, in the colon tissue. All three gingerols also reduced the DSS-induced serum levels of the pro-inflammatory cytokines TNF and IL-1β (67).

A subsequent study further explored the mechanism behind the anti-inflammatory effects of 6-gingerol in the DSS-induced mouse colitis model (68). Orally-administered 6-gingerol significantly decreased the DSS-induced weight loss of mice. It was demonstrated that 6-gingerol decreased IL-17 levels, while it increased IL-10 levels both in the serum and bowel tissues of DSS-treated mice. Moreover, 6-gingerol inhibited the DSS-induced phosphorylation of IκB and the phosphorylation and nuclear translocation of p65 in the bowel tissue (68).

Another study investigated the efficacy of orally-administered 6-shogaol-loaded nanoparticles in the mouse model of DSS-induced colitis (69). Similar to gingerols, 6-shogaol also alleviated colitis symptoms and accelerated wound repair. In addition, 6-shogaol significantly decreased the levels of TNF, IL-6, IL-1β and iNOS, while it increased the expression of the anti-inflammatory genes HO-1 and NRF2 in colon tissues of DSS-treated mice (69).

A recent study compared the anti-colitis efficacy of 6-, 8-, 10-gingerols and shogaols in the DSS-induced colitis mouse model (70). All components were able to prevent DSS-induced weight loss, colon length reduction and IL-1β, IL-6 and IFN-γ serum levels in DSS-treated mice. Among the ginger compounds, 8-, and 10- shogaols, which were also able to significantly decrease DSS-induced serum levels of TNF, showed the greatest efficacy compared to the other compounds. 6-, 8-, and 10-gingerols and 6-, 8-, and 10-shogaols also downregulated iNOS and COX-2 protein levels and significantly reduced phosphorylation of NF-κB in colonic tissue. Interestingly, 10-shogaol was found to be the most potent in its ability to block iNOS and COX-2 expression and NF-κB activity. It was also the most effective in increasing the expression of tight junction proteins, thus the intestinal integrity of DSS-treated mice. The study concluded that 8-, and 10-shogaols are highly efficient in their ability to suppress colitis symptoms and inflammation, thus may serve as better candidates for the treatment of colitis than the corresponding gingerols.

Multiple sclerosis (MS) is a T cell-mediated autoimmune disease of the central nervous system that is characterized by immune-mediated demyelination in the spinal cord and cerebral cortex (71). The neuroprotective effects of ginger phenolics were extensively studied in animal models of neurotoxicity and brain damage [reviewed in (19)]; however, so far only two studies investigated the immunomodulatory activity of gingerols and shogaols in experimental autoimmune encephalomyelitis (EAE), the mouse model of MS. The first study showed that 6-gingerol decreased the inflammatory infiltration and demyelination in the white matter of spinal cord of EAE mice (53). 6-gingerol-treated mice had lower numbers of inflammatory cells including DCs and T cells in the spleen. Further, splenocytes from 6-gingerol treated mice produced significantly lower levels of IL-17 and GM-CSF indicating that 6-gingerol inhibited Th17 cell polarization *in vivo* (53). In addition, 6-gingerol lowered the percentage of leukocytes such as CD11c+MHC II+ DCs, CD45+CD11b+ monocytes and Th17 cells in the central nervous system (CNS) of EAE mice. This suggests that 6-gingerol inhibits inflammatory cell infiltration in the CNS. The next study investigated whether 6-shogaol or its metabolite, 6-paradol could ameliorate EAE symptoms and inflammation (72). Administration of 6-shogaol or 6-paradol significantly reduced the clinical signs of the disease. Histological analysis showed that both components decreased demyelination, cell accumulation and TNF expression in the spinal cord of EAE mice. In addition, 6-shogaol and 6-paradol markedly reduced astrogliosis and microglial activation of EAE mice. These findings suggest that the neuroprotective effects of 6-gingerol and 6-paradol in EAE might be associated to the dampened inflammatory responses in the CNS (72).

The protective role of 6-gingerol was also investigated in the context of systemic autoimmune diseases such as systemic lupus erythematosus (SLE) and rheumatoid arthritis (RA). SLE affects almost every organ in the body and is characterized by the appearance of anti-nuclear autoantibodies and circulating immune complexes. In a lupus mouse model 6-gingerol administration significantly reduced serum levels of anti-dsDNA, cell-free DNA and also MPO-DNA complexes, the latter of which serves as a marker of NET formation (47). 6-gingerol also greatly reduced the serum levels of the pro-inflammatory cytokines TNF and IFN-γ. In line with that, 6-gingerol suppressed netosis and thus large-vein thrombosis in a mouse model of antiphospholipid syndrome (APS) as well. Pharmacokinetic studies further revealed that 6-gingerol accumulated in neutrophils, even while its plasma level was dropping (47). These results are in line with previous reports showing that ginger phenolics are converted into glucuronide conjugates and thus are rapidly cleared from the plasma. In tissues, these conjugated forms are then reconverted into their free active form by specific enzymes such as β-glucuronidase (73, 74).

RA is also a systemic chronic immune-mediated disorder, which is characterized by synovial inflammation, joint damage, loss of function and deformities. IL-17 is one of the key cytokines in promoting inflammation and thus cartilage damage in RA (75). Therefore, in a recent study, 315 natural extracts were tested in their ability to inhibit IL-17-induced IL-6 production by synovial cells (76). Among these extracts, dried ginger and in particular its specific component 8-shogaol showed the highest inhibitory activity against IL-17-mediated IL-6 secretion by synovial cells and macrophages. Thereafter, the anti-arthritic potency of 8-shogaol was investigated in adjuvant induced arthritis (AIA), a rat model of RA (76). Treatment with 8-shogaol reduced paw thickness and weight loss of AIA rats and improved their walking performance as well. Furthermore, 8-shogaol treatment decreased bone erosion and cellular infiltration into the joints of AIA rats. Upon 8-shogaol treatment the levels of TNF, IL-6 and IL-8 were markedly downregulated both in the serum and synovial tissues of AIA rats. These data suggest that 8-shogaol has the potential to ameliorate disease severity and inflammation in RA (76).

The above data indicate that the bioactive compounds of ginger exert a general anti-inflammatory effect by efficiently decreasing immune reactions in a number of autoimmune and inflammatory disease models, thus it is plausible that ginger-derived phytochemicals might serve as supplementary agents in the therapy of these diseases. The effects of the major ginger phenolics in animal models of autoimmune and autoinflammatory diseases are included in **Table 5**.

**Table 5:** *In vivo* effects of ginger phenolics in animal models of autoinflammatory and autoimmune diseases.

Compounds	Models	Administration routes	Observed effects	Potential mechanisms	Ref.
6-, 8-, and 10-gingerols	DSS-induced rat colitis model	intraperitoneal	• attenuated DSS-induced symptoms of colitis • accelerated mucosal damage healing • decreased neutrophil infiltration in the colon • reduced TNF and IL-1β serum levels	• increased SOD activity • decreased MPO activity	(67)
6-gingerol	DSS-induced mouse colitis model	oral	• decreased DSS-induced weight loss • decreased IL-17 levels and increased IL-10 levels both in serum and bowel tissue	• inhibited IκB phosphorylation • suppressed NF-κB nuclear translocation in the bowel tissue	(68)
6-shogaol	DSS-induced mouse colitis model	oral	• alleviated colitis symptoms • accelerated wound repair • decreased the levels of TNF, IL-6, IL-1β and iNOS	• increased HO-1 and NRF2 expression in the colon tissues	(69)
6-, 8-, 10-gingerols and 6-, 8-, 10-shogaols	DSS-induced mouse colitis model	oral	• prevent DSS-induced weight loss, colon length reduction • reduced IL-1β, IL-6 and IFN-γ serum levels • downregulated iNOS and COX-2 levels	• reduced NF-κB phosphorylation	(70)
6-gingerol	EAE mice	intraperitoneal	• decreased inflammatory cell infiltration and demyelination in spinal cord and CNS • lowered number of inflammatory cells in the spleen • inhibited Th17 polarization	–	(53)
6-shogaol, 6-paradol	EAE mice	oral	• decreased demyelination, cell accumulation and TNF expression in spinal cord • reduced astrogliosis and microglial activation	–	(72)
6-gingerol	lupus and APS mouse model	intraperitoneal	• reduced serum level of anti-dsDNA, cell-free DNA, MPO-DNA complexes, TNF, IFN-γ • suppressed netosis • inhibited thrombosis in APS	–	(47)
8-shogaol	AIA rats	intraperitoneal	• reduced paw thickness and weight loss • improved walking • decreased bone erosion and cellular infiltration to the joints • reduced TNF, IL-6 and IL-8 levels in serum and synovial tissues	–	(76)

## 4 Proposed mechanism of action of gingerols and shogaols

As we have introduced above ginger constituents are able to interfere with common inflammatory signaling pathways including the NF-κB, MAPK and PI3K/Akt/mTOR cascades in different immune cell types; however, the previously introduced *in vitro* studies did not, or rarely revealed the mechanism behind the anti-inflammatory actions of ginger phenolics. Nevertheless, mounting evidence, mostly from *in vivo* studies and experiments with non-immune cell types, indicates that ginger constituents might affect the inflammatory signaling pathways by targeting different upstream regulatory molecules such as AMPK, NRF2, or PPARγ. Recent publications also suggest that these compounds might exert their actions through binding to the TRPV1 ion channel. In this chapter we summarize our current knowledge about the possible mechanisms of immunomodulation by different ginger phenolics.

### 4.1 The mechanism of action of ginger phenolics through AMPK activation

AMPK is a central regulator of diverse physiological and metabolic processes, which can be dysregulated in pathological conditions, such as cancer, obesity or chronic inflammatory diseases (77). Generally, AMPK blocks mTORC1 activation by phosphorylating two of its regulatory proteins. On the one hand AMPK phosphorylates and thereby increases the activity of Tuberous Sclerosis Complex 2 (TSC2), an upstream inhibitor of mTORC1. On the other hand, it phosphorylates Raptor, a scaffold protein of mTORC1 that also leads to mTORC1 inactivation and consequently to the suppression of anabolic processes and inflammation (78). Accumulating evidence suggests a negative correlation between AMPK activity and inflammation (77). Chronic inflammation is associated with reduced AMPK activity and thereby aberrant mTOR activity. Thus, targeting AMPK might serve as a potential therapeutic strategy for treating inflammatory-related diseases (79). Many phytochemicals such as resveratrol, quercetin, and curcumin act as natural activators of AMPK (80). Recent studies also suggest that ginger phytochemicals might also exert their anti-inflammatory effects on immune cells via suppressing mTOR and increasing AMPK activity.

Initially, studies reported that 6-shogaol inhibit inflammation by suppressing the phosphorylation of key mTOR pathway components. First it was demonstrated that 6-shogaol inhibited the LPS-induced phosphorylation and activation of NF-κB by interfering with the PI3K/Akt and MAPK signaling pathways in murine macrophages (34). Thereafter, it was shown that 6-shogaol inhibited TNF-induced disassembly of tight junctions via inhibition of NF-κB and PI3K/Akt in the human colonic epithelial cell line HT-29/B6 (81). Another study further demonstrated that 6-gingerol suppressed LPS-induced microglia activation by down-regulating the Akt/mTOR pathway activity (38).

Later it was also demonstrated that 6-gingerol indirectly suppress mTOR activity by promoting the activation of AMPK. First it was reported that ginger extract restored the high fat diet (HFD)-induced downregulation of AMPK activity in the liver of rats (82). Another study presented that 6-gingerol attenuates hepatic steatosis, inflammation and oxidative stress in HFD-fed mice via activating AMPK (83). In particular, intragastrical administration of 6-gingerol decreased the concentration of TNF, IL-6 and ROS, while it increased the phosphorylation level of liver kinase B1 (LKB1) and AMPK in the liver of HFD-fed mice. LKB1 is one of the master upstream kinases of AMPK, which forms a trimeric complex with two other proteins and then phosphorylates and activates AMPK. The authors suggest that 6-gingerol enhanced the stability of the LKB1 complex that consequently increased LKB1/AMPK pathway activity in the liver of mice. These results were also strengthened by *in vitro* experiments using palmitic acid-treated HepG2 cells, in which 6-gingerol reduced lipid accumulation and oxidative stress by increasing LKB1 complex stability and thus LKB1/AMPK pathway activity (83).

Recent observations further suggest that the anti-diabetic and anti-cancer efficacy of ginger phenolics are also associated with their ability to induce AMPK activation. It was reported that 6-shogaol and 6-paradol promote glucose utilization in mouse adipocytes that could be attributed to the increased activity of AMPK (84). Furthermore, 6-gingerol was found to suppress oral cancer cell growth by inducing the activation of AMPK and blocking mTOR pathway activity (85), while 10-gingerol was shown to inhibit the proliferation and migration of vascular smooth muscle cells by AMPK activation (86). The latter study also demonstrated a stable binding between 10-gingerol and AMPK by molecular docking studies and surface plasmon resonance imaging analysis. This was the first study showing a direct interaction between 10-gingerol and AMPK that proposed 10-gingerol as a natural agonist of AMPK. Nevertheless, there are no available data on the binding affinity of other ginger phytochemicals to AMPK.

Although, some reports demonstrated that gingerols and shogaols decrease PI3K/Akt/mTOR signaling pathway activity in immune cells, the regulatory role of AMPK activity was not investigated in these studies. We have recently reported that 6-shogaol was able to inhibit mTORC1 signaling through upregulating AMPK activity in moDCs, while 6-gingerol was not able to do so (54). Nevertheless, further studies are needed to reveal whether the anti-inflammatory activities of gingerols and shogaols could be generally linked to increased AMPK activity.

**Figure 3:**
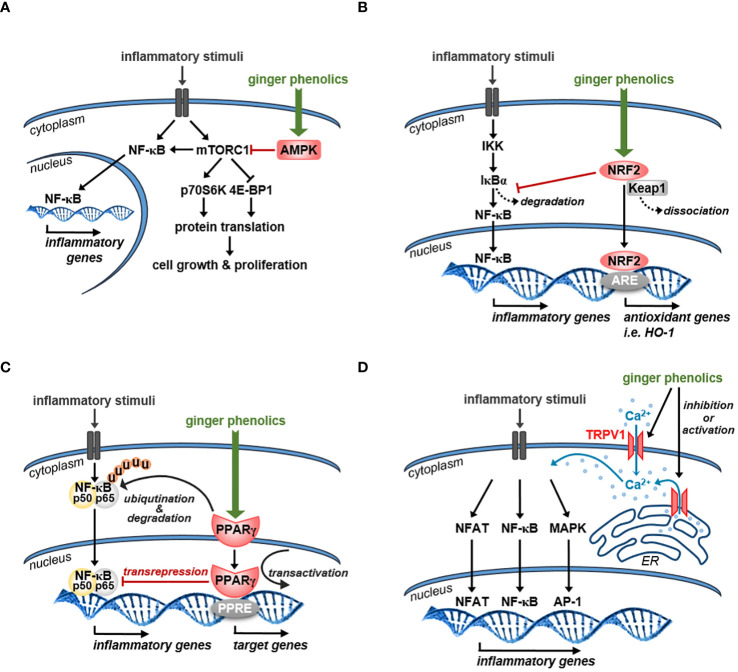
Schematic representation of the possible mechanism of action of ginger phenolics in immune cells **(A)** Ginger phenolics suppress mTORC1 functionality by increasing AMPK activity. Generally, mTORC1 supports protein synthesis and thus cell growth and proliferation by direct phosphorylation of p70S6 kinase (p70S6K) and eukaryotic initiation factor 4E-binding protein 1 (4E-BP1). In immune cells mTORC1 is also involved in NF-κB activation and its target gene expression. **(B)** Ginger-derived bioactive compounds might also exert their anti-inflammatory effects by activating NRF2, which induces the expression of an array of antioxidant and cytoprotective genes. NRF2 also inhibits NF-κB activity by preventing IκBα proteasomal degradation and thus blocks the nuclear translocation of NF-κB. **(C)** Ginger phenolics might also exert their anti-inflammatory effects by activating PPARγ. By interacting with p65 NF-κB, PPARγ can induce its ubiquitination and proteolytic degradation. Stimulation of PPAR-γ limits NF-kB dependent gene transcription through transrepression as well. Moreover, PPARγ supports the expression of inhibitory proteins such as IκBα via transactivation. **(D)** Ginger constituents can directly interact with TRPV1 channels and might act as either TRPV1 agonists or antagonists. Generally, TRPV1 mediated Ca^2+^ influx facilitates the activation of different transcription factors that might be facilitated or inhibited by ginger-derived bioactive compounds. *NFAT: nuclear factor of activated T cells, PPRE: PPAR response elements*.

**Figure 3A** shows how the activation of AMPK by the major ginger phenolics might affect the mTOR and thus the NF-κB signaling pathways.

### 4.2 Anti-inflammatory actions of ginger phenolics through NRF2 regulation

NRF2 is a transcription factor that plays a pivotal role in the regulation of antioxidant and anti-inflammatory responses (87). Besides inducing the expression of an array of enzymes involved in antioxidant defense and detoxification, it is able to negatively regulate NF-κB activity by multiple mechanisms. For instance, by inducing antioxidant enzymes NRF2 decreases ROS levels that consequently inhibit oxidative stress-mediated NF-κB activation. NRF2 is also able to prevent IκBα proteasomal degradation and thus the nuclear translocation of NF-κB. In addition, a series of *in vitro* and *in vivo* studies indicated that NRF2-mediated HO-1 expression substantially contributes to the anti-inflammatory activity of NRF2. As an important antioxidant enzyme HO-1 catalyzes the degradation of toxic free heme that leads to the release of anti-inflammatory products, such as carbon monoxide and biliverdin (88). Beyond its enzymatic functions, HO-1 exerts non-canonical, signaling functions, through which HO-1 is able to control essential cellular signaling processes such as gene expression, protein translation, and DNA repair mechanisms (89).

In the last few years many studies proposed that ginger-derived bioactive compounds exert their anti-inflammatory effects by activating the NRF2/HO-1 axis. A study suggest that this is especially true for shogaols, which in contrast to gingerols, bear an α,β-unsaturated carbonyl group in their side chain (90). Molecular modeling revealed that 6-shogaol is able to increase HO-1 levels through a Michael reaction between its α, β-unsaturated carbonyl group and Kelch-like ECH-associated protein 1 (Keap1), which acts as a negative regulator of NRF2. This interaction initiates conformational changes in Keap1 that allows the release of NRF2 from Keap1 leading to its nuclear translocation. In the nucleus NRF2 binds to the antioxidant response element (ARE) and induces the expression of various antioxidant and anti-inflammatory genes. In that particular study, 6-shogaol was found to upregulate the protein levels of HO-1 and suppress thrombin-induced NO release in rat microglia, while 6-gingerol was not able to do so (90). 6-shogaol also increased the nuclear import of NRF2 in BV2 microglia and significantly enhanced the HO-1 mRNA level in primary-cultured microglia (90). Similarly, we have recently demonstrated that 6-shogaol increases the expression and nuclear translocation of NRF2 and enhances the protein levels of HO-1, while 6-gingerol does not affect that in human moDCs (54). In an *in vivo* mouse ICH model, 6-shogaol increased striatal HO-1 protein levels and rescued neuron loss (90). Another study demonstrated that 6-shogaol protects against renal ischemia-reperfusion (I/R) injury as well. 6-shogaol pretreatment significantly decreased the expression of various pro-inflammatory cytokines and chemokines in mice subjected to renal I/R. Mechanistically, 6-shogaol reduced kidney inflammation by attenuating NF-κB activation and inducing HO-1 expression (91). In the same year it was published that 6-shogaol prevented UVB-induced inflammation and oxidative stress through modulating NRF2 signaling in human epidermal keratinocytes (HaCaT cells) (92). 6-shogaol significantly decreased the UVB-triggered expression of IL-6, TNF and IL-10, while reduced the phosphorylation of ERK, JNK and p38 MAPKs in human epidermal keratinocytes. The authors further demonstrated that 6-shogaol prevented UVB-induced depletion of NRF2 and elevated HO-1 protein levels in HaCaT cells. In another *in vitro* model, 6-shogaol treatment increased the expression of HO-1 in LPS-stimulated human umbilical vein endothelial cells (HUVECs) as well (93). Similar to 6-shogaol, 6-gingerol was also able to activate the NRF2/Keap1 signaling pathway. It was demonstrated that 6-gingerol significantly reduced the expression of Keap1 and increased that of NRF2 in the nuclear fraction of buccal pouch tissues of 7,12‐dimethylbenz(a)anthracened (DMBA)-treated hamsters (94). By doing so, 6-gingerol prevented buccal pouch carcinogenesis through inhibiting the expression of inflammatory and cell proliferation markers such as IL‐6, TNF‐α, IL‐1β, iNOS, COX‐2 and cyclin D, while inducing pro-apoptotic markers such as Bax in DMBA-induced hamsters. These findings were further strengthened by another study showing that 6-gingerol also alleviates sepsis-induced liver injury through activating the NRF2 pathway (65). Pre-treatment with 6-gingerol attenuated cecal ligation and puncture (CLP)-induced hepatic inflammation and injury by increasing the protein levels of NRF2 and HO-1 in liver homogenates of C57BL/6 mice. These results were further confirmed by an *in vitro* model. 6-gingerol suppressed ATP-induced pyroptosis, IL-1β and caspase-1 secretion and ROS production by preventing the downregulation of NRF2 and HO-1 protein levels in LPS-primed RAW 264.7 cells (65). Furthermore, it was shown that 6-gingerol decreased cardiac injury via the NRF2/HO-1 pathway in both mouse and cell models of diabetic cardiomyopathy (DCM) (95). In the mouse heart 6-gingerol inhibited the expression of ferroptosis-related proteins, while enhanced the expression of anti-ferroptosis-related proteins. In addition, 6-gingerol treatment decreased the levels of IL-1β, IL-6, and TNF-α in serum and heart tissues of diabetic mice. In H9c2 cardiac cells, 6-gingerol also inhibited ferroptosis and inflammation evoked by palmitic acid and high glucose administration. Most importantly, 6-gingerol enhanced the NRF2 and HO-1 protein levels both in the mouse and cell models of DCM. Hence, the authors suggested that 6-gingerol might protect against DCM by inhibiting ferroptosis and inflammation via activating the NRF2/HO-1 pathway (95). A novel study suggests that 6-gingerol attenuates sepsis-induced acute lung injury by suppressing NLRP3 inflammasome activation via NRF2 activation (43). In LPS-induced rats, 6-gingerol repressed the expression of various oxidative stress markers, inflammatory cytokines and NLRP3 inflammasome components in the lung tissues and inhibited the infiltration of inflammatory cells into the lungs. In addition, 6-gingerol prevented the LPS-mediated downregulation of NRF2 and HO-1 levels in the lung tissues of rats. Intraperitoneal injection of ML385, an NRF2 inhibitor, reversed the protective effect of 6-gingerol against LPS-induced oxidative stress and inflammation suggesting that 6-gingerol exerts its anti-inflammatory effects through activating the NRF2/HO-1 axis (43). Similar to 6-gingerol, zingerone, a less-studied natural compound of ginger, also reduced organ injury through activating the NRF2 signaling pathway in a CLP-induced sepsis model (49). In particular, administration of zingerone alleviated ROS accumulation and systematic inflammation in septic mice and inhibited NET formation both *in vivo* and *in vitro*. The results further suggest that zingerone attenuates NET formation and inflammation via NRF2-dependent ROS inhibition (49).

As the above data indicate many studies have demonstrated that the major bioactive compounds of ginger are able to activate the NRF2/HO-1 axis that seems to greatly contribute to their anti-inflammatory effects. Lately, emerging evidence indicate a cooperation between AMPK and NRF2 signaling as well. In particular, AMPK might function as a positive upstream regulator of the NRF2/HO-1 system and thus can lead to the transactivation of specific target genes (96). Interestingly, the AMPK-mediated enhancement of the NRF2/HO-1 response does not seem to depend on mTOR inhibition (97). In our studies, we found that 6-shogaol is able to increase the activity of both AMPK and NRF2 in human DCs (54). Nevertheless, it needs to be further elucidated whether these components contribute separately to the ant-inflammatory potential of 6-shogaol or AMPK acts as an upstream regulator of NRF2.

It is also plausible that certain phytochemicals might control NRF2 activity through the E3 ubiquitin ligase tripartite motif-containing 29 (TRIM29), also known as ataxia telangiectasia group D complementing (ATDC). Recent evidences indicate that TRIM29 plays a versatile role in orchestrating inflammatory responses. In alveolar macrophages, TRIM29 negatively regulates the LPS-induced pro-inflammatory cytokine production by inducing the ubiquitination and degradation of NF-κB essential modulator (NEMO) (98). In mice, TRIM29 controls pro-inflammatory cytokine production by acting as a central regulator of protein kinase RNA-like endoplasmic reticulum kinase (PERK)-driven endoplasmic reticulum (ER) stress response (99). Mechanistic studies have also shown that TRIM29 is also able to bind to and sequester KEAP1, thereby preventing NRF2 from proteasomal degradation in pancreatic cancer cells (100). To the best of our knowledge, it has never been investigated whether plant-derived compounds are able to control TRIM29; however, as the previous reports suggest TRIM29 acts as a multifunctional protein, and thus might regulate immune responses by various mechanisms. In the future, it could also be explored whether phytochemicals control NRF2 activity through TRIM29 that might help to better understand the mechanism behind their anti-inflammatory actions.

**Figure 3B** represents how ginger phenolics might exert their anti-inflammatory effects via activating the NRF2 signaling pathway.

### 4.3 Mode of action of ginger-derived compounds through PPARy regulation

PPARy is a member of the nuclear receptor superfamily that is widely expressed in immune and endothelial cells (101). It is able to regulate various biological functions, such as inflammatory responses and lipid metabolism. Most importantly, PPARγ can suppress inflammation by promoting the inactivation of NF-κB through direct or indirect mechanisms. PPARγ can cause ubiquitination and proteolytic degradation of p65 NF-κB, and it can also promote the expression of inhibitory proteins such as IκBα or HO-1. Many dietary phytochemicals target PPARγ (102), however, only a few studies investigated whether 6-gingerol and 6-shogaol act as PPARγ agonists.

In rats subjected to ventilator-induced lung injury, GW9662, a selective PPARγ inhibitor abolished the protective effect of 6-gingerol (103). In particular, the 6-gingerol-mediated decrease in pro-inflammatory cytokine release, neutrophil accumulation and oxidative stress in lung tissues of rats challenged with mechanical ventilation was partially reversed by the inhibition of PPARγ (103). 6-shogaol was also suggested to exert its anti-inflammatory effects in LPS-activated BV2 microglia by activating PPARγ (37). In these cells 6-shogaol significantly reduced LPS-induced NF-κB activation, pro-inflammatory cytokine and PGE_2_ release that could be reversed by GW9662 treatment. In contrast, another study presented that GW9662 could not reverse the inhibitory effect of 6-shogaol on TNF-induced disassembly of tight junctions in human colonic HT-29/B6 cells (81).

Although data on the relation of ginger phytochemicals to PPARγ activity is scarce and contradictory, we cannot exclude the possibility that the anti-inflammatory effects of gingerols and shogaols are at least partially dependent on PPARγ activity.

**Figure 3C** shows that 6-gingerol and 6-shogaol might inhibit the NF-κB pathway through activating PPARγ.

### 4.4 The mechanism of action of ginger-derived phytochemicals through targeting TRPV1

TRPV1 is primarily expressed in sensory neurons and plays an essential role in heat sensation and nociception. Accumulating data suggests that TRPV1 is also expressed in various mammalian immune cells, especially in macrophages, neutrophils, DCs and T cells, where it modulates various functions such as cytokine release, migration or phagocytic activity (104). Nevertheless, exploring the role of TRPV1 on immune cells is hindered by the sometimes-contradictory results gained from human and animal cells. Specifically, TRPV1 activation promotes the maturation and activation of murine DCs (105), while inhibits the differentiation, maturation, phagocytosis and cytokine secretion of human moDCs (106).

The major pungent compounds of ginger share certain structural characteristics with capsaicin, the main pungent constituent found in chili pepper, and the prototypic agonist of TRPV1 (107). In particular, gingerol, shogaol and zingerone contain the same vanillyl head group as capsaicin; therefore, these ginger constituents can directly activate TRPV1 channels, though with a much lower potency than capsaicin (108). Specifically, a study showed that while capsaicin shows strong potency on TRPV1, 6-shogaol and 6-gingerol have presented only moderate potency on the channel (109). It was further demonstrated that 6‐shogaol, 6‐gingerol, and zingerone bind to the same ligand‐binding pocket in TRPV1 channels as capsaicin; however, the distinct structural features in their tails cause large differences in their potency (110). 6‐shogaol is the most similar to capsaicin regarding its molecular structure. The aliphatic tail of 6-shogaol similar in length to that of capsaicin, and 6-shogaol has the same functional groups, which are able to form hydrogen bonds with the TRPV1 channels. capsaicin. The major difference between 6-shogaol and capsaicin is that the C═C bond is located at the tip of the tail in capsaicin, whereas it is at base of the tail in 6-shogaol. Although, shifting the C═C bond limits the rotational freedom of the tail of 6-shogaol, 6-shogaol represents the strongest agonist of TRPV1 among the ginger compounds (110). In comparison to 6‐shogaol, 6‐gingerol is slightly weaker in its potency to induce TRPV1 activity. This might be due to the presence of a hydroxyl group instead of a double bond in the tail of 6-gingerol that reduces its lengths. Finally, zingerone is a weak agonist for TRPV1 channels that might be explained by the shortness of its aliphatic tail. Altogether, the study suggests that the length of the aliphatic tail, the presence or absence of the hydroxyl group, and the position of the C═C bond in the tail all might influence the ability of ginger phenolics to bind and activate TRPV1 (110).

In a rat model of I/R injury 6-gingerol was found to inhibit NLRP3-mediated inflammation and neuronal apoptosis, while it upregulated autophagy (111). The mechanistic findings indicate that 6-gingerol exerts anti-apoptotic and anti-inflammatory effects during cerebral I/R injury via dissociating TRPV1 from Fas-associated factor 1 (FAF1), which is able to modulate the sensitivity and thus the activity of TRPV1 to various stimuli.

The ligand-induced activation of TRPV1 by ginger compounds in non-neuronal cell types is poorly characterized. So far, only one study investigated whether the ligand-induced activation of TRPV1 by 6-gingerol is able to affect the functionality of neutrophils (50). The analyses showed that 6-gingerol increased intracellular Ca^2+^ concentrations and the expression of common surface markers such as CD11b and CD66b in neutrophils. Moreover, 6-gingerol enhanced fMLP-stimulated CXCL8 secretion and ROS production that could be reversed by pharmacological inhibition of TRPV1. Here the authors applied 6-gingerol at a low dose of 50 nM, which is a dietary relevant concentration that could be reached in blood plasma after consumption of 1 liter of ginger tea (50). In contrast, 50 µM of geranylacetone, a natural sesquiterpenoid, acting as a TRPV1 agonist inhibited fMLP-induced migration of neutrophils and CXCL8-induced intracellular Ca^2+^ mobilization (112). These observations suggest that lower concentrations of TRPV1 ligands might induce immune cell functions, while higher TRPV1 ligand concentrations in the µM range rather inhibit immune responses. Therefore, further studies are needed to reveal the exact mode of action of ginger phenolics on TRPV1.

**Figure 3D** shows that acting through TRPV1, various ginger phenolics might affect the activity of various signaling pathways.

## 5 Concluding remarks and future perspectives

In this review, our goal was to compile the most relevant findings on the immunomodulatory actions of the major ginger phenolics. We mainly focused on the anti-inflammatory effects of these compounds on different immune cells and presented a variety of studies, which demonstrated the possible mode of their anti-inflammatory actions. Though, comparative studies are sparse, most of the findings indicate that 6-shogaol exhibit a stronger anti-inflammatory and antioxidant capacity than 6-gingerol that might be attributed to the presence of the α,β-unsaturated ketone moiety in its skeleton. Although the exact mechanism of action of ginger-derived bioactive compounds are not yet fully elucidated, data show that the application of these compounds results in the successful inhibition of common signaling pathways such as the NF-κB and PI3K/Akt/mTOR signaling cascades that consequently leads to the suppression of inflammatory responses by immune cells. Interestingly, contradictory results were obtained regarding the action of gingerols and shogaols on the activity of MAPK signaling components; however, the reason for the varying results has not been revealed yet. Research data summarized above further suggest that ginger phytochemicals exert their pleiotropic effects via targeting multiple regulatory molecules including AMPK, PPARγ, NRF2 and also TRPV1. In addition, recent *in silico* molecular docking and molecular dynamics stimulation studies revealed that 6-gingerol has a binding affinity towards COX-1/2 and 5- lipoxygenase, thus it has the potential to directly target various enzymes associated with inflammation (113).

Ginger has been used traditionally as an herbal medicine for the treatment of many maladies, and due to their potent immunomodulatory capacity several reports proposed ginger-derived bioactive compounds as candidates for the management and prevention of autoimmune diseases. Nevertheless, clinical studies are lacking, probably due to the poor water solubility and adsorption of ginger phenolics. Among the organic solvents, ethanol has been proposed as an optimal solvent since it is able to retain the bioactivity of ginger compounds (114, 115). Pharmacokinetic studies also revealed that once ginger is consumed gingerols and shogaols are absorbed quickly and are majorly present in the form of water soluble glucuronide conjugates in the human plasma (74, 116). Although the level of ginger phenolics and their metabolites is low and drops quickly in the serum, experiments with mice demonstrated that gingerols and shogaols may reach higher concentrations in tissues in comparison to the serum levels due to the accumulation of free and conjugated forms of ginger phenolics in various organs (73).

Nevertheless, further studies are needed to increase the solubility, stability, half-life time and thus the therapeutic efficiency of ginger phenolics. Recently, more and more projects are focusing on the development of novel formulation techniques and drug delivery systems to increase the efficacy and to reduce the toxicity of the bioactive compounds of ginger (104, 117–120). Current studies indicate that nanoformulation, ensuring a sustained release of ginger constituents, might provide an effective delivery system. Possible nanotechnological approaches include carriers, such as nanovesicles, exosome-like nanoparticles, nanostructure lipid carrier, and emulsions (20). Optimization of nano-drug delivery systems for the targeted delivery and controlled release of ginger phenolics is still in its infancy; however, it is feasible that in the future ginger supplements might provide an alternative or most probably a complementary therapeutic approach to treat various inflammatory and autoimmune disorders.

## Author contributions

KP: Writing – original draft, Writing – review & editing. AS: Writing – original draft, Writing – review & editing. TF: Writing – original draft, Writing – review & editing.
